# Secretome of Stressed Peripheral Blood Mononuclear Cells Alters Transcriptome Signature in Heart, Liver, and Spleen after an Experimental Acute Myocardial Infarction: An In Silico Analysis

**DOI:** 10.3390/biology11010116

**Published:** 2022-01-13

**Authors:** Caterina Selina Mildner, Dragan Copic, Matthias Zimmermann, Michael Lichtenauer, Martin Direder, Katharina Klas, Daniel Bormann, Alfred Gugerell, Bernhard Moser, Konrad Hoetzenecker, Lucian Beer, Mariann Gyöngyösi, Hendrik Jan Ankersmit, Maria Laggner

**Affiliations:** 1 Department of Thoracic Surgery, Medical University of Vienna, 1090 Vienna, Austria; cate.mildner@gmail.com (C.S.M.); dragan.copic@meduniwien.ac.at (D.C.); martin.direder@meduniwien.ac.at (M.D.); katharina.klas@meduniwien.ac.at (K.K.); daniel.bormann@meduniwien.ac.at (D.B.); alfred.gugerell@meduniwien.ac.at (A.G.); bernhard.moser@meduniwien.ac.at (B.M.); konrad.hoetzenecker@meduniwien.ac.at (K.H.); 2 Applied Immunology Laboratory, Medical University of Vienna, 1090 Vienna, Austria; 3 Aposcience AG, 1200 Vienna, Austria; 4 Christian Doppler Laboratory for Cardiac and Thoracic Diagnosis and Regeneration, Department of Thoracic Surgery, Medical University of Vienna, 1090 Vienna, Austria; matthias.zimmermann@meduniwien.ac.at; 5 Department of Oral and Maxillofacial Surgery, Medical University of Vienna, 1090 Vienna, Austria; 6 Department of Internal Medicine II, Division of Cardiology, Paracelsus Medical University of Salzburg, 5020 Salzburg, Austria; m.lichtenauer@salk.at; 7 Department of Biomedical Imaging and Image-Guided Therapy, Medical University of Vienna, 1090 Vienna, Austria; lucian.beer@meduniwien.ac.at; 8 Department of Medicine II, Division of Cardiology, Medical University of Vienna, 1090 Vienna, Austria; mariann.gyongyosi@meduniwien.ac.at

**Keywords:** therapeutic secretome, PBMC secretome, acute myocardial infarction, regenerative medicine, ischemia/reperfusion, paracrine action

## Abstract

**Simple Summary:**

Acute myocardial infarction is characterized by impaired coronary blood flow, which leads to cardiac ischemia and, ultimately, compromised heart function. Damage and cellular responses are not limited to the non-perfused area, but rather affect the entire heart, as well as distal organs, such as the liver and spleen. We found that the therapeutic secretome of stressed white blood cells improved short-term and long-term cardiac performance in a porcine infarction model. In order to unravel the molecular events governing secretome-mediated tissue regeneration, we performed transcriptional analyses of the non-perfused, transition, and perfused heart, as well as the liver and spleen 24 h after myocardial infarction. We observed a highly tissue-specific effect of the secretome and, except for the transition zone, a uniform downregulation of pro-inflammatory factors and pathways. Simultaneously, the secretome strongly promoted the expression of genes that are essential for heart function in the non-perfused area. In the liver and spleen, different metabolic processes were induced. Together, our data suggest several plausible mechanisms by which the secretome improves heart function after cardiac ischemia. Deepening our understanding of the molecular processes identified here might uncover further pharmacologic strategies aiming at delimiting adverse cardiac remodeling and sequelae after myocardial infarction.

**Abstract:**

Acute myocardial infarction (AMI) is a result of cardiac non-perfusion and leads to cardiomyocyte necrosis, inflammation, and compromised cardiac performance. Here, we showed that the secretome of γ-irradiated peripheral blood mononuclear cells (PBMCsec) improved heart function in a porcine AMI model and displayed beneficial long- and short-term effects. As an AMI is known to strongly affect gene regulation of the ischemia non-affected heart muscle and distal organs, we employed a transcriptomics approach to further study the immediate molecular events orchestrated using the PBMCsec in myocardium, liver, and spleen 24 h post ischemia. In the infarcted area, the PBMCsec mainly induced genes that were essential for cardiomyocyte function and simultaneously downregulated pro-inflammatory genes. Interestingly, genes associated with pro-inflammatory processes were activated in the transition zone, while being downregulated in the remote zone. In the liver, we observed a pronounced inhibition of immune responses using the PBMCsec, while genes involved in urea and tricarboxylic cycles were induced. The spleen displayed elevated lipid metabolism and reduced immunological processes. Together, our study suggested several types of pharmacodynamics by which the PBMCsec conferred immediate cardioprotection. Furthermore, our data supported the assumption that an AMI significantly affects distal organs, suggesting that a holistic treatment of an AMI, as achieved by PBMCsec, might be highly beneficial.

## 1. Introduction

According to the World Health Organization, ischemic heart disease (IHD) represents the leading cause of death worldwide, accounting for 16% of the world’s total deaths, with 8.9 million cases in 2019 [1,2]. The most prevalent symptomatic manifestations and complications of IHD are acute myocardial infarction (AMI), heart failure, cardiac arrhythmias, and cardiac arrest [3]. AMI pathophysiology is characterized by decreased coronary blood flow, which results in insufficient oxygen supply and, henceforth, myocardial ischemia [4]. Oxygen deprivation induces necrosis in cardiomyocytes, connective tissues, and blood vessels, which represents an early hallmark of an AMI [5]. Necrotic cells, in turn, initiate an innate immune response via several pro-inflammatory signaling pathways [6]. As a result, leukocytes are readily recruited to the border zone, contributing to the aggravation of local cardiotoxic effects [7]. In addition, neutrophils have been shown to infiltrate the infarcted area, augmenting tissue damage by releasing extracellular traps and, thereby, further obstructing the vasculature [8,9,10]. Eventually, an AMI results in tissue fibrosis and scarring, leading to compromised cardiac performance. A previous study showed that an AMI not only modulates the ischemic areas but also affects the ischemia non-affected areas of the myocardium, which represent the non-necrotic remote zone with sufficient oxygen supply [11]. Furthermore, an AMI induces a condition of systemic inflammation [12]. In detail, several genes associated with inflammation were upregulated in the liver and spleen following an AMI, such as *KLF4*, *NFKB1*, *STAT1*, and *STAT3*. Furthermore, chemokine signaling pathways were induced by AMI in the liver. Several investigations showed that myocardial infarction elicits a global response, influencing the bone marrow [13], liver [14], kidney [15], and spleen [16]. The AMI-related molecular events prevailing in the infarcted area, non-affected myocardium, liver, and spleen were already studied in detail [12]. These systemic effects suggest the need for a holistic treatment approach for AMI.

In regenerative medicine, the therapeutic potential of cell-derived secretomes has become increasingly recognized. Our group demonstrated strong tissue-regenerative properties of irradiated peripheral blood mononuclear cells (PBMCs) in the ischemic conditions of experimental AMI [17,18], and irradiation-induced necroptosis was found to be indispensable for this effect [19]. In-depth analyses identified numerous biologically active constituents present in the secretome obtained from γ-irradiated PBMCs (PBMCsec), such as proteins, extracellular vesicles, and lipids [20,21,22,23,24]. Functional studies unveiled a diverse action spectrum of these pleiotropic substances, including anti-inflammatory [23,25], tissue-regenerative [20], anti-microbial [26], vasodilatory [27], and pro-angiogenic [28] properties. During the course of seminal research works, the therapeutic efficacy of the PBMCsec was successfully demonstrated in various ischemic indications, including AMI [22,29], chronic ischemic left ventricular dysfunction [30], and cerebral ischemia [31], but also numerous other conditions, such as autoimmune myocarditis [32], acute spinal cord injury [33], burn injury [34], diabetic wounds [24], and dendritic cell-mediated skin inflammation [21].

Intravenous administration of the PBMCsec exerts immediate and long-term cardio-protective effects [22,27]. Short-term benefits manifested within 24 h post AMI, including diminished necrosis, reduced troponin I release, as well as induction of the vasodilatory mediators nitric oxide (NO) and prostacyclin (PGI_2_) [22,27]. In addition, thrombolysis in myocardial infarction (TIMI) frame counts was reduced, while myocardial blush grade was improved using the PBMCsec [27]. Furthermore, the incidence of rhythmological occurrences was remarkably lower within 60 min after reperfusion when applying the PBMCsec [27]. Beneficial long-term effects of the PBMCsec were observed 30 days after ischemia and were evidenced by reduced infarct area and markedly improved cardiac performance [22]. No transcriptomics data on the effect of PBMCsec treatment on a healthy heart, spleen, or liver are available.

Toxicological safety and tolerability of the PBMCsec were already demonstrated (clinicaltrials.gov identifiers NCT02284360 and NCT04277598, accessed on 21 September 2021) [35,36,37]. Currently, the PBMCsec is being tested in a phase II trial to treat diabetic foot ulcers (NCT04277598, EudraCT number 2018-001653-27).

**Figure 1:**
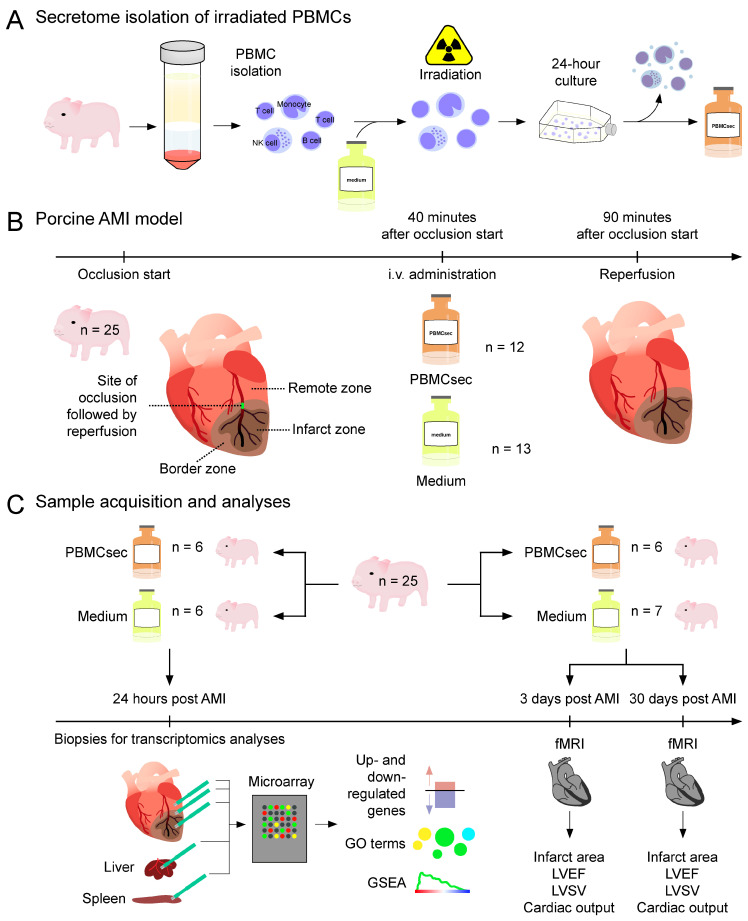
Background and experimental approach. (**A**) Porcine PBMCs were enriched, irradiated, and cultured for 24 h. Cells and cellular debris were removed and the supernatant containing the secretome was lyophilized. (**B**) Reperfused AMI was induced via balloon occlusion of the left anterior descending coronary artery (LAD) for a total duration of 90 min, followed by reperfusion. Forty minutes after starting the balloon inflation, the PBMCsec (n = 12 animals) or medium used for the PBMC culture (n = 13) were injected over 25 min. (**C**) For the in silico analyses, biopsies of infarcted, transition, and non-infarcted myocardium, as well as liver and spleen of the PBMCsec-treated (n = 6) and medium-treated pigs (n = 6), were obtained 24 h post AMI. Transcriptional analyses included the identification of up- and downregulated genes, Gene Ontology (GO) term analysis, and gene set enrichment analysis (GSEA). Cardiac performance was determined using functional magnetic resonance imaging (fMRI) 3 days and 30 days after the AMI and assessed in terms of infarct area, LVEF, LVSV, and cardiac output. fMRIs of the PBMCsec-treated (n = 6) and medium-treated (n = 7) pigs was performed 3 days and 30 days after the AMI. AMI: acute myocardial infarction, GO: gene ontology, GSEA: gene set enrichment analysis, i.v.: intravenous, fMRI: functional magnetic resonance imaging, LVEF: left ventricular ejection fraction, LVSV: left ventricular stroke volume, PBMCsec: secretome of stressed porcine peripheral blood mononuclear cells.

Despite these preceding studies, the exact molecular processes governing the immediate actions of the PBMCsec during ischemic conditions remain elusive. Furthermore, potential systemic effects on distal organs have not been investigated to date. As several effects were observed within the first 24 h, we sought to determine the short-term transcriptional programs orchestrated by PBMCsec treatment in the infarcted, transition, and remote zones of the myocardium, as well as in the liver and spleen 24 h after occlusion (Figure 1). These insights might help unravel alternative therapeutic targets to ameliorate the post-AMI outcome.

## 2. Materials and Methods

### 2.1. Ethical Statement

Animal experiments were carried out in accordance with the “Position of the American Heart Association on Research Animal Use”, and this study was approved by the Ethics Committee on Animal Experimentation of the University of Kaposvar, Kaposvar, Hungary (votes 246/002/SOM/2006 and MAB-28-2005). Animal experiments were performed at the animal testing facility of the University of Kaposvar equipped with a magnetic resonance imaging device suitable for large animals. All remaining experiments were performed at the Medical University of Vienna.

### 2.2. Generation of PBMCsec

Allogeneic porcine PBMCsec was generated from healthy female Large White pigs (*Sus domesticus*), as described previously [22]. Briefly, blood was obtained from pigs not used for AMI experiments via heart puncture and PBMCs were enriched using Ficoll-Paque PLUS (GE Healthcare, Chicago, IL, USA) density gradient centrifugation. PBMCs were exposed to 60 Gy γ-irradiation (IBL 437C, Isotopen Diagnostik CIS GmbH, Dreieich, Germany) and cultured in phenol red-free CellGenix GMP DC medium (CellGenix, Freiburg, Germany) for 24 ± 2 h. Cells and cellular debris were removed via centrifugation and supernatants containing the secretome were dialyzed against ammonium acetate, passed through 0.22 µm filters, and lyophilized in aliquots to avoid repeated freeze–thaw cycles. Lyophilized DC medium without cells served as the control. Lyophilisates were stored below −70 °C for less than 1 month. Before use, lyophilisates were brought to room temperature, resuspended in 0.9% NaCl, and immediately administered.

### 2.3. Porcine Closed-Chest Occlusion–Reperfusion Infarction Model and PBMCsec Treatment

For experimental AMI, a closed-chest occlusion–reperfusion AMI infarction model was used [12,22]. Female Large Whites were 90 days old at the start of the experiments and displayed an average weight of 31.86 ± 9.1 kg. In total, 25 animals were used. Pigs were sedated with 12 mg/kg ketamine hydrochloride, 1 mg/kg xylazine, and 40 µg/kg atropine and received 200 IU/kg unfractionated heparin. Animals were intratracheally intubated, and general anesthesia was maintained during the reperfused AMI. The right femoral artery was prepared and an introducer was placed. After diagnostic coronary angiography, a Maverick balloon catheter (3 mm diameter and 15 mm length; Boston Scientific, Natick, MA, USA) was inserted into the LAD. The balloon was inflated for 90 min, and 40 min after starting the occlusion, allogeneic porcine PBMCsec (lyophilized supernatants of 10^9^ porcine PBMCs resuspended in 250 mL saline) or 250 mL medium were injected intravenously over 25 min. Ninety minutes after the occlusion, the balloon was deflated and reperfusion was re-established. Animals received 75 mg clopidogrel and 100 mg acetylsalicylic acid as postoperative medication. None of the animals died due to the experimental interventions.

Experimental AMI and PBMCsec treatments were performed for two sets of animals. For transcriptional analyses, the first set of animals (n = 6 PBMCsec-treated animals and n = 6 medium-treated animals) was euthanized 24 h post AMI. In a second set of animals (n = 6 PBMCsec-treated and n = 7 medium-treated), cardiac performance was monitored using functional magnetic resonance imaging (fMRI) [12,22]. Infarct area, LVEF, LVSV, and cardiac output were determined in the second set of animals using fMRI 3 days and 30 days after the AMI.

### 2.4. RNA Isolation and Microarray Gene Expression Analysis

Identification of the three different myocardial areas was performed as established previously [12,22]. The fibrous scar area was considered to be the infarct core zone, and the myocardium proximal to the LAD occlusion segment was considered to be the non-infarcted remote zone. The transition zone in between was defined as the border zone. Tissues were isolated by 6 mm biopsy punches and washed in saline. Biopsies were lysed in RNA later stabilization solution (Invitrogen, Thermo Fisher Scientific, Waltham, MA, USA). Tissues were homogenized (Precellys, Bertin Instruments, Rockville, MD, USA) and total RNA was isolated by RNeasy Mini Kit (Qiagen, Hilden, Germany). RNA quality was assessed using a 2100 Bioanalyzer (Agilent, Santa Clara, CA, USA), and all samples displayed RNA integrity numbers > 8. One hundred nanograms were used for gene expression profiling (Miltenyi Biotec, Bergisch-Gladbach, Nordrhein-Westfalen, Germany) on Whole Porcine Genome Oligo Microarrays (Agilent, Santa Clara, CA, USA). Microarrays were carried out by Miltenyi Biotec. Differentially expressed genes (DEGs) were defined as genes displaying more than a 1.5-fold change of average expression values and were used for the enrichment analysis with Cytoscape (v3.8.5) [38] using the ClueGO (v2.5.7) plug-in [39]. Biological process, immune system process, molecular function, and KEGG were selected to identify pathways and ontologies associated with DEGs. The *p*-values were determined using ClueGO and Bonferroni step-down correction was used to correct for multiple comparisons [39]. Gene set enrichment analysis (GSEA) of DEGs was performed in R (The R Foundation, Vienna, Austria) using the clusterprofiler package [40,41].

### 2.5. Statistical Analyses

GraphPad Prism software (version 8.0.2, GraphPad Software Inc., La Jolla, CA, USA) was used for statistical evaluation and to generate heatmaps. Infarct area, LVEF, LVSV, and cardiac output were compared using a two-tailed *t*-test for unpaired samples. The Gaussian distribution of the data was tested using graphical means (histograms) and the F-test used to compare variances displayed no significant differences in any parameter at any time point. Data are presented as arithmetic mean ± standard deviation.

## 3. Results

### 3.1. PBMCsec Improved Cardiac Regeneration after AMI

**Figure 2:**
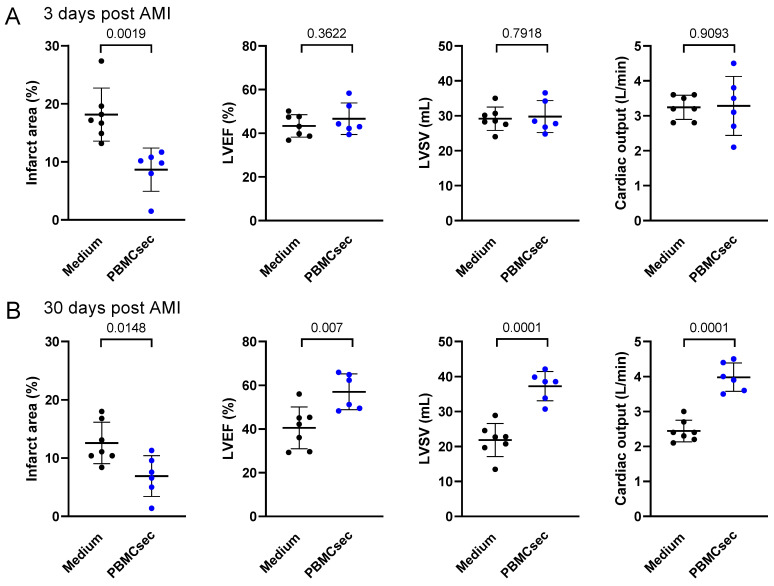
The PBMCsec promoted cardiac regeneration post AMI. Infarct area, LVEF, LVSV, and cardiac output of the medium-treated pigs (n = 7) and PBMCsec-treated pigs (n = 6) were assessed (**A**) 3 days and (**B**) 30 days after a LAD balloon occlusion. A two-sided *t*-test for unpaired samples was used to compare the groups. Numbers indicate *p*-values. LVEF: left ventricular ejection fraction, LVSV: left ventricular stroke volume.

Myocardial ischemia was induced in a porcine closed-chest AMI model via 90 min transient balloon occlusion of the left anterior descending artery (LAD) and the PBMCsec or medium were injected intravenously starting 40 min after the occlusion. Examination on day 3 following the AMI revealed a significant reduction in the relative infarction area with a trend toward improved left ventricular performance with the PBMCsec (Figure 2). This was even more pronounced on day 30, where the PBMCsec-treated animals showed significantly improved LVEF, LVSV, and cardiac output. These data indicated that the intravenous PBMCsec administration 40 min after the start of an arterial occlusion exerted beneficial short- and long-term effects to promote post-AMI cardiac regeneration.

### 3.2. PBMCsec Induced Genes Associated with Cardiac Muscle Contraction and Curbed Inflammation within the Infarcted Zone 24 h after AMI

**Figure 3:**
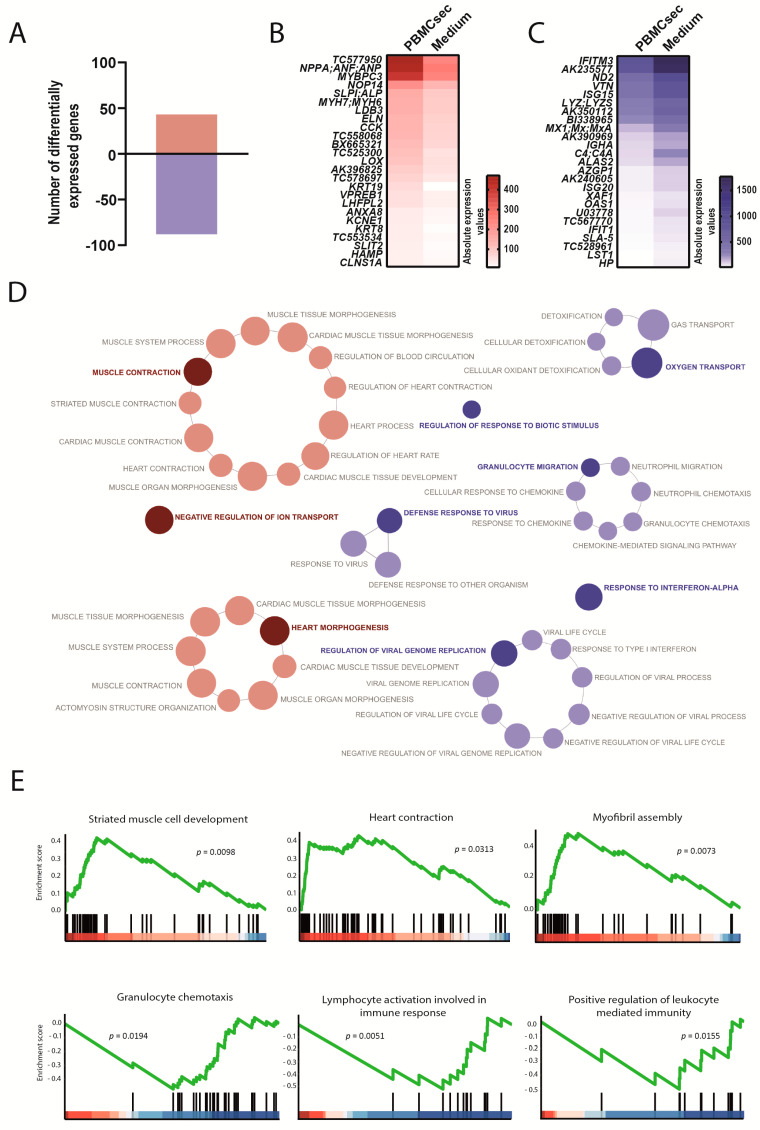
Transcriptional changes induced by the PBMCsec in the infarcted area. (**A**) Total numbers of differentially expressed genes. Red and blue indicate up- and downregulated genes, respectively, when comparing the PBMCsec *versus* the medium. The average fold change of n = 6 animals per group is shown. Absolute expression values of genes (**B**) up- and (**C**) downregulated by the PBMCsec compared with the medium. Colors indicate expression values. (**D**) GO terms associated with up- (red) and downregulated (blue) genes. Each circle represents one term. Circle sizes indicate *p*-values. Overarching terms within a group are highlighted in bold. (**E**) GSEA of up- (upper panel) and downregulated (lower panel) genes by the PBMCsec.

Since several short-term (within 24 h) effects of the PBMCsec in the infarcted heart were reported [22,27], we proceeded to investigate the transcriptional programs coordinated by the PBMCsec 24 h after an AMI. Within the infarct zone, we detected 131 differentially expressed genes between PBMCsec and the medium, whereby 43 and 88 genes were up- and downregulated, respectively (Figure 3A, Supplementary File S1). Among the most upregulated genes, we found natriuretic peptide A (*NPPA*), the cardiac myosin binding protein c (*MYBPC3*), as well as cardiac α and β myosin heavy chain 6 and 7 (*MYH6* and *MYH7*) (Figure 3B). Simultaneously, PBMCsec downregulated genes involved in inflammatory processes, such as interferon-induced transmembrane protein 3 (*IFITM3*), interferon-induced protein with tetratricopeptide repeats 1 (*IFIT1*), interferon-stimulated gene 15 (*ISG15*) lysozyme (*LYZ*), and MHC class I antigen 5 (*SLA-5*) (Figure 3C). Gene ontology (GO) enrichment analysis revealed that the genes upregulated by the PBMCsec were associated with muscle contraction, heart morphogenesis, and ion transport (Figure 3D, Supplementary File S2). Conversely, the PBMCsec induced downregulation of genes implicated in granulocyte migration, responses to interferon-α, and viral response (Figure 3D), indicating that the PBMCsec exerted anti-inflammatory effects in the infarct zone. We furthermore performed GSEA and observed that the PBMCsec induced genes associated with cardiomyocyte function whilst concomitantly downregulating leukocyte chemotaxis and activation (Figure 3E), corroborating findings obtained by the GO term analysis.

### 3.3. PBMCsec Promoted Pro-Inflammatory Processes in the Transition Zone between Perfused and Non-Perfused Areas 24 h after AMI

**Figure 4:**
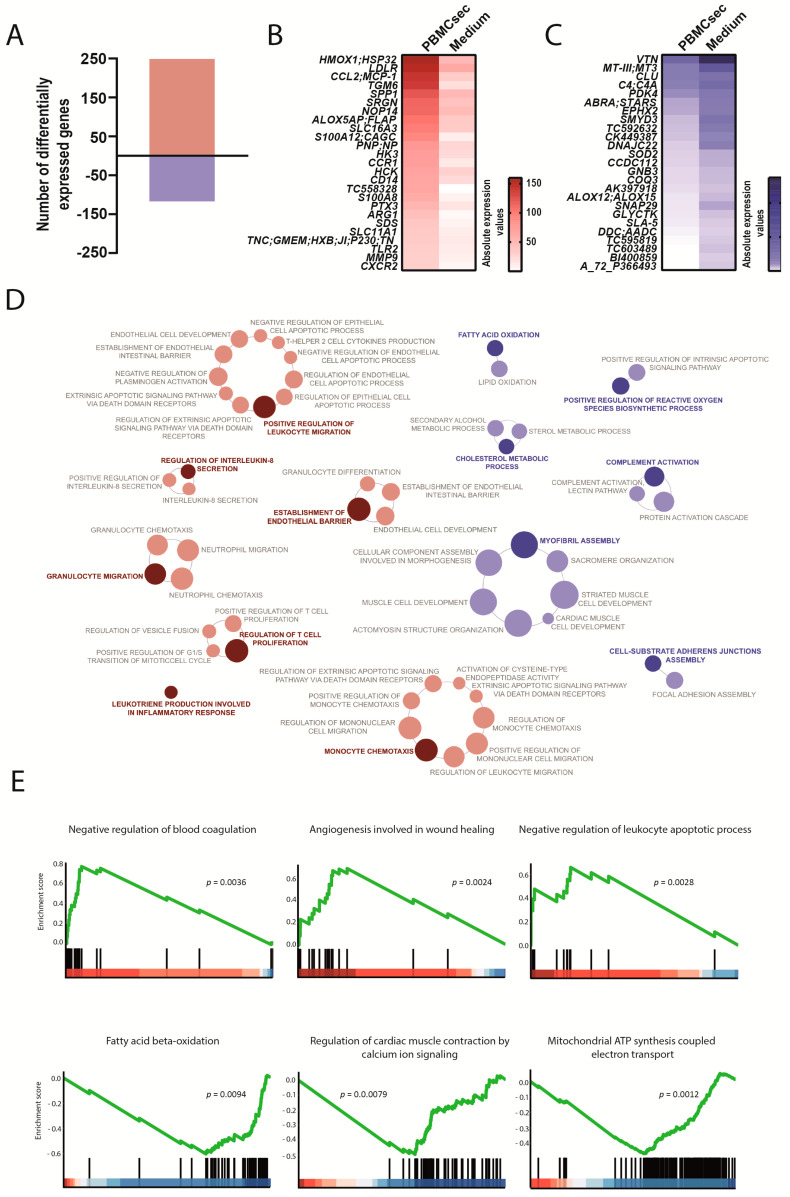
Transcriptional changes induced by the PBMCsec in the border zone between infarcted and remote myocardium. (**A**) Total numbers of differentially expressed genes. Red and blue indicate up- and downregulated genes, respectively, when comparing the PBMCsec *versus* the medium. The average fold change of n = 6 animals per group is shown. Absolute expression values of genes (**B**) up- and (**C**) downregulated by the PBMCsec compared with the medium. Colors indicate expression values. (**D**) GO terms associated with up- (red) and downregulated (blue) genes. Each circle represents one term. Circle sizes indicate *p*-values. Overarching terms within a group are highlighted in bold. (**E**) GSEA of up- (upper panel) and downregulated (lower panel) genes by the PBMCsec.

We next assessed the transcriptional profile of the border zone and observed a distinct gene expression pattern compared with the infarct zone. A total of 366 genes were differentially expressed, with 249 up- and 117 downregulated genes when comparing the PBMCsec with the medium (Figure 4A, Supplementary File S1). Interestingly, we observed a specific and differential regulation of immunological responses in the transitional zone by the PBMCsec compared with the non-perfused area. Several genes involved in pro-inflammatory processes, such as C-C motif chemokine ligand 2 (*CCL2*), C-C motif chemokine receptor 1 (*CCR1*), cluster of differentiation 14 (*CD14*), and C-X-C motif chemokine receptor 2 (*CXCR2*), were upregulated by the PBMCsec. Similarly, the calgranulin genes *S100A8* and *S100A12*, as well as toll-like receptor 2 (*TLR2*), were induced by the PBMCsec (Figure 4B). Furthermore, arginase 1 (*ARG1*) was upregulated in the border zones of the PBMCsec-treated pigs. The anti-oxidant defense gene heme oxygenase 1 (*HMOX1*) and the anti-fibrotic gene matrix metallopeptidase 9 (*MMP9*) were strongly induced by the PBMCsec. Among genes required for leukotriene biosynthesis, arachidonate 5-lipoxygenase-activating protein (*ALOX5AP*) was found upregulated by the PBMCsec (Figure 4B), while *ALOX12* and *ALOX15* were downregulated (Figure 4C). Moreover, superoxide dismutase 2 (*SOD2*), *SLA-5*, and complement factor 4a (*C4A*) were downregulated by the PBMCsec in the border zone. In accordance with the upregulation of genes involved in pro-inflammatory responses, several immunological processes, such as leukocyte and granulocyte migration, monocyte chemotaxis, T cell proliferation, IL-8 secretion, leukotriene production, and endothelial barrier establishment, were potentially activated by the PBMCsec (Figure 4D, Supplementary File S2). Pathways associated with downregulated genes in the border zone were reactive oxygen species biosynthesis pathways, fatty acid oxidation, complement system, and myofibril assembly (Figure 4D). The establishment of the endothelial barrier identified via GO enrichment analysis was confirmed with angiogenesis involved in wound healing using GSEA (Figure 4E). Fatty acid oxidation and diminished myofibril assembly were corroborated using GSEA with downregulated pathways, including fatty acid beta-oxidation and cardiac muscle contraction. GSEA further revealed the inhibition of blood coagulation, leukocyte apoptosis, and respiratory chain assembly (Figure 4E). These data suggested that the PBMCsec induced a specific, local gene regulatory program depending on the tissue location relative to the ischemic event.

### 3.4. PBMCsec Suppressed Inflammation in the Remote Zone 24 h after AMI

**Figure 5:**
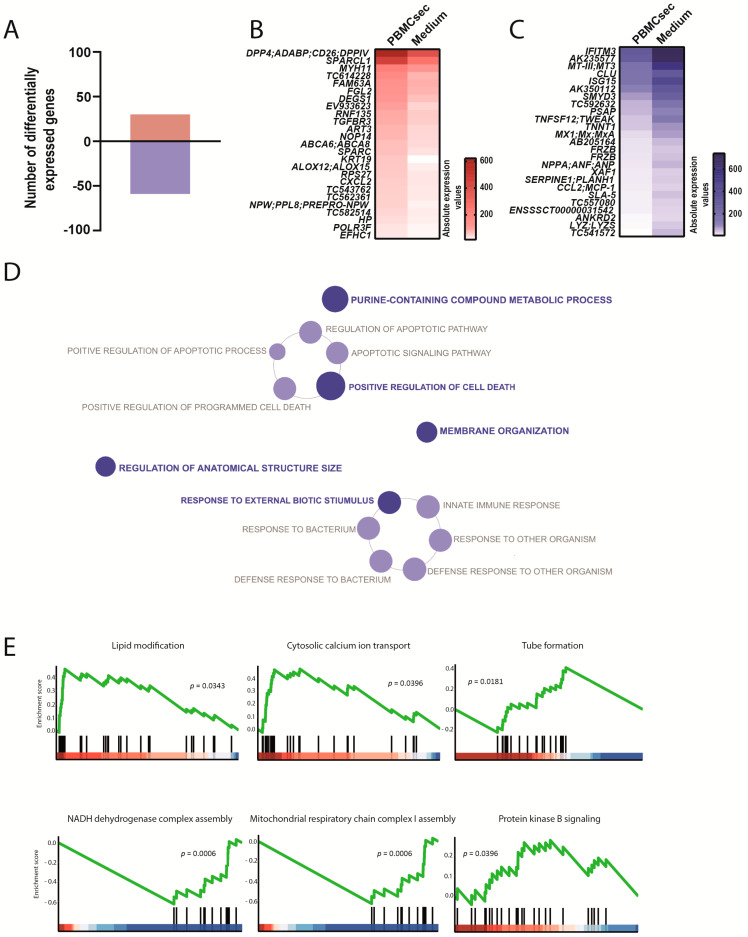
Transcriptional changes induced by the PBMCsec in the intact myocardium. (**A**) Total numbers of differentially expressed genes. Red and blue indicate up- and downregulated genes, respectively, when comparing the PBMCsec *versus* the medium. The average fold change of n = 6 animals per group is shown. Absolute expression values of genes (**B**) up- and (**C**) downregulated by the PBMCsec compared with the medium. Colors indicate expression values. (**D**) GO terms associated with downregulated genes. Each circle represents one term. Circle sizes indicate *p*-values. Overarching terms within a group are highlighted in bold. (**E**) GSEA of up- (upper panel and protein kinase B in the lower panel) and downregulated (two terms on the lower left) genes by the PBMCsec.

Next, we determined the gene expression signatures of the remote zone, which represented perfused, functional myocardium in proximity to the infarcted area. We observed a total of 89 differently expressed genes, whereby 30 genes were up- and 59 genes were downregulated by the PBMCsec compared with the medium (Figure 5A, Supplementary File S1). Among the most significantly regulated genes, we found an increased expression of dipeptidyl peptidase-4 (*DPP4*), myosin heavy chain 11 (*MYH11*), *ALOX12*, and *ALOX15*. In addition, fibrinogen-like 2 (*FGL2*), transforming growth factor beta receptor 3 (*TGFBR3*), and C-X-C motif chemokine ligand 2 (*CXCL2*) were strongly induced (Figure 5B). In contrast, tumor necrosis factor superfamily member 12 (*TNFSF12*), *NPPA*, *IFTM3*, *SERPINE* 1, *CLL2*, and *SLA-5* were downregulated by the PBMCsec (Figure 5C), indicating PBMCsec-induced anti-inflammatory properties. As only very few genes were upregulated, GO enrichment analysis only revealed terms for downregulated genes, which included the positive regulation of cell death, response to external biotic stimulus, and membrane organization (Figure 5D, Supplementary File S2). GSEA revealed lipid modification, calcium transport, tube formation, and protein kinase B signaling as upregulated functions, while NADH dehydrogenase and respiratory chain assembly were suppressed by the PBMCsec (Figure 5E). These data further substantiated the concept of highly tissue-specific gene regulation by the PBMCsec.

### 3.5. Systemic Effects of PBMCsec on Distal Organs 24 h following AMI

**Figure 6:**
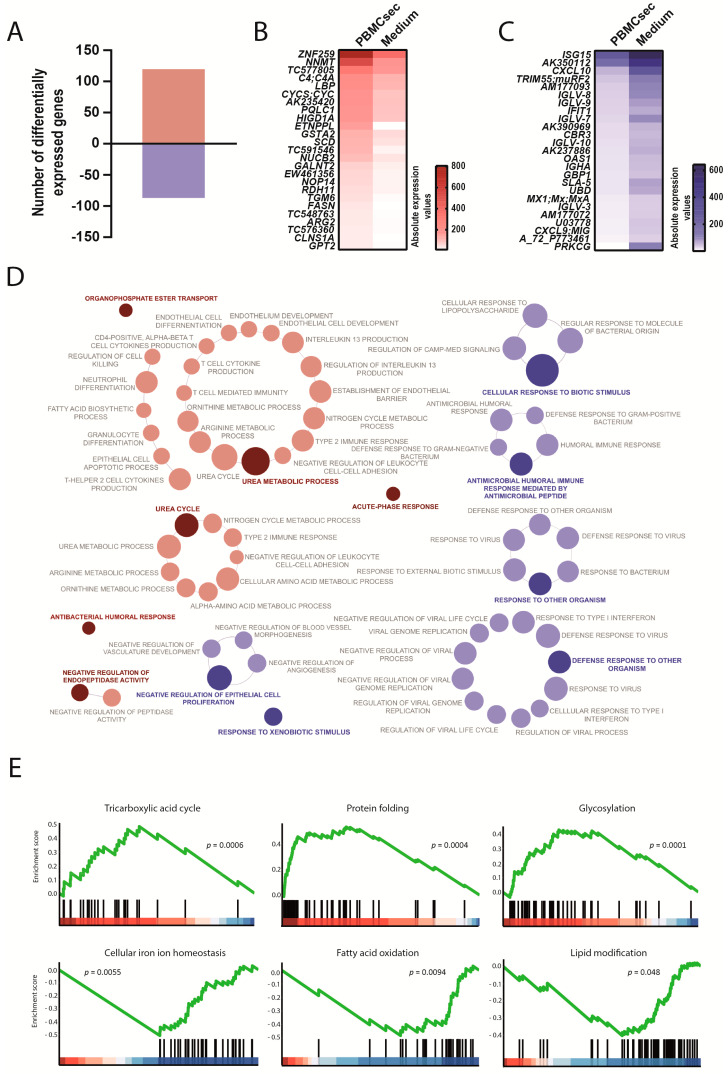
Transcriptional changes induced by the PBMCsec in the liver. (**A**) Total numbers of differentially expressed genes. Red and blue indicate up- and downregulated genes, respectively, when comparing the PBMCsec *versus* the medium. The average fold change of n = 6 animals per group is shown. Absolute expression values of genes (**B**) up- and (**C**) downregulated by the PBMCsec compared with the medium. Colors indicate expression values. (**D**) GO terms associated with up- (red) and downregulated (blue) genes. Each circle represents one term. Circle sizes indicate *p*-values. Overarching terms within a group are highlighted in bold. (**E**) GSEA of up- (upper panel and protein glycosylation in lower panel) and downregulated (two terms on lower left) genes by the PBMCsec.

To assess the potential systemic effects of the PBMCsec in the context of ischemic events, we analyzed the transcriptional differences in the liver and spleen between the PBMCsec and the medium 24 h after AMI. In the liver, a total of 207 genes were differentially expressed and PBMCsec induced up- and downregulation of 120 and 87 genes, respectively (Figure 6A, Supplementary File S1). Compared with the medium, the PBMCsec promoted the expression of *C4A*, lipopolysaccharide-binding protein (*LBP*), hypoxia-inducible domain family member 1A (*HIGD1A*), and arginase 2 (*ARG2*). Furthermore, upregulation of stearoyl-CoA desaturase (*SCD*) and fatty acid synthase (*FASN*) was observed (Figure 6B). Simultaneously, downregulated genes included oligoadenylate synthase 1 (*OAS1*) and ubiquitin D (*UBD*). In addition, we discovered the decreased expression of C-X-C motif chemokine 10 (*CXCL10*) and *SLA-5* (Figure 6C). In the liver, the genes upregulated by the PBMCsec were involved in immune system pathways, such as acute-phase response and antibacterial humeral response. Furthermore, the PBMCsec induced genes involved in the negative regulation of endopeptidase activity and urea metabolic processes. Intriguingly, biological processes, such as response to (xeno)biotic stimulus and antimicrobial humoral immune response mediated by antimicrobial peptide, were downregulated by the PBMCsec in the liver (Figure 6D, Supplementary File S2). GSEA identified the tricaboxylic acid cycle, protein folding, and protein glycosylation as gene sets enriched in upregulated genes, while cellular iron homeostasis and fatty acid oxidation were downregulated by the PBMCsec (Figure 6E).

**Figure 7:**
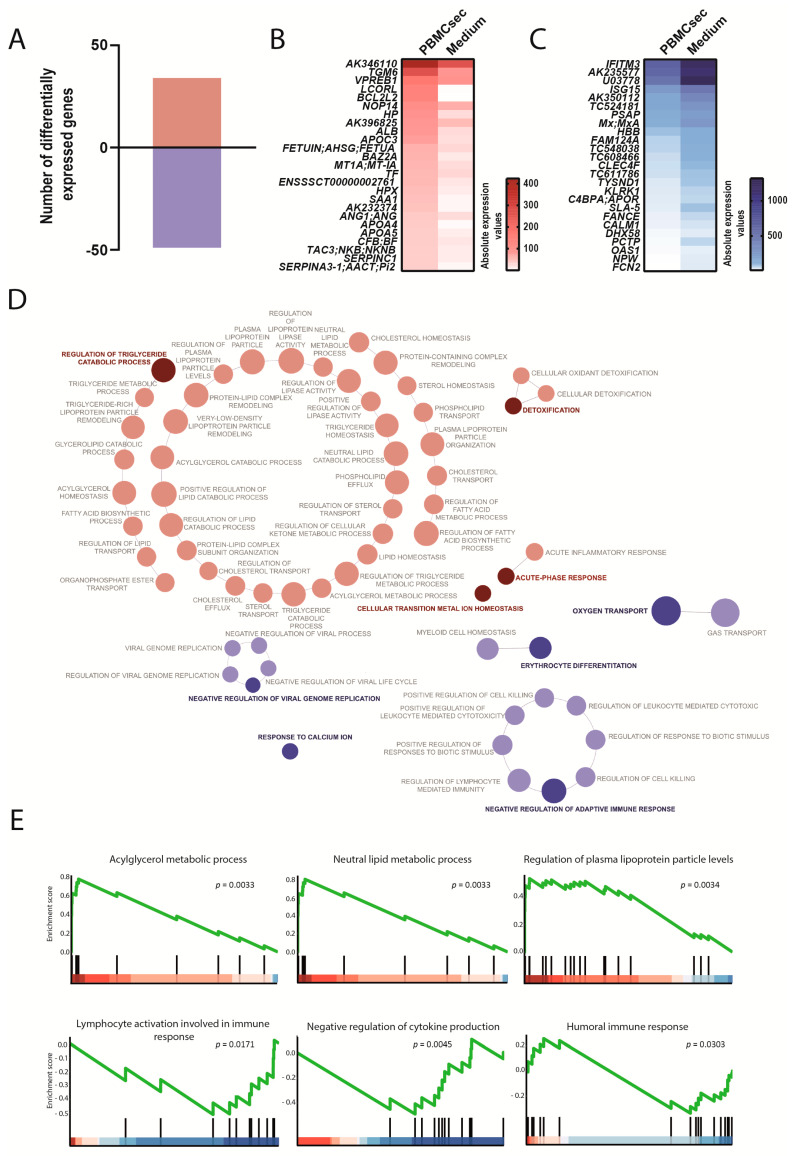
Transcriptional changes induced by the PBMCsec in the spleen. (**A**) Total numbers of differentially expressed genes. Red and blue indicate up- and downregulated genes, respectively, when comparing the PBMCsec *versus* the medium. The average fold change of n = 6 animals per group is shown. Absolute expression values of genes (**B**) up- and (**C**) downregulated by the PBMCsec compared with the medium. Colors indicate expression values. (**D**) GO terms associated with up- (red) and downregulated (blue) genes. Each circle represents one term. Circle sizes indicate *p*-values. Overarching terms within a group are highlighted in bold. (**E**) GSEA of up- (upper panel) and downregulated (lower panel) genes by the PBMCsec.

Lastly, the distal effects of the PBMCsec on the spleen were determined. We observed 34 up- and 49 downregulated genes after the application of the PBMCsec (Figure 7A, Supplementary File S1). Expressions of angiopoietin 1 (*ANG1*), BCL2-like 2 (*BCL2L2*), and V-set pre-B cell surrogate light chain 1 (*VPREB1*) were induced and the upregulation of apolipoprotein C3, A4, and A5 (*APOC3*, *APOA4*, and *APOA5*) was observed (Figure 7B). In addition, the PBMCsec inhibited the expression of *SLA-5*, *OAS1*, ISG15 ubiquitin-like modifier (*ISG15*), and ficolin-2 (*FCN2*) (Figure 7C). In the spleen, genes implicated in the regulation of the triglyceride catabolic process were strongly induced in the PBMCsec-treated pigs. Furthermore, detoxification and acute-phase response pathways were upregulated. Conversely, genes downregulated by the PBMCsec were involved in the negative regulation of adaptive immune response, oxygen transport, erythrocyte differentiation, negative regulation of viral genome replication, and response to calcium ion (Figure 7D, Supplementary File S2). GSEA unveiled that the PBMCsec induced genes associated with lipid metabolic processes, plasma lipoprotein levels, and acylglycerol metabolism (Figure 7E). While lymphocyte activation and the humoral immune response were downregulated, negative regulation of cytokine production was also reduced by the PBMCsec.

## 4. Discussion

In the last few decades, several key players implicated in the cardiac response to ischemia have been identified [7,11,12,42,43]. Intravenous and intramyocardial PBMCsec administration provides therapeutic short- and long-term benefits in the treatment of AMI and in preventing cardiac fibrosis and scarring [18,22,27,30]. In the current study, we meticulously delineated the early molecular programs by which the PBMCsec attenuated cardiac damage and inflammation and furthermore promoted tissue regeneration following an AMI, both on a local and systemic level 24 h after the AMI.

Inflammation is an indispensable hurdle in the course of wound repair and post-AMI tissue regeneration [44]. Ischemic cardiomyocytes undergo necrosis, thereby releasing their intracellular content and triggering an inflammatory cascade via innate mechanisms [6]. Recruited immune cells mediate phagocytic clearance of necrotic cardiomyocytes. However, an excessive immunological response results in (secondary) tissue damage of viable cardiomyocytes [44,45,46]. Considering the accentuated tissue damage caused by exaggerated immune cell activity, immuno-suppressants were regarded as a promising treatment approach to attenuate cardiac injury secondary to an AMI. However, anti-inflammatory agents failed to improve post-AMI outcomes in humans [44,47]. In fact, interfering with inflammatory processes governing post-ischemia tissue regeneration delays the clearance of apoptotic and necrotic cells [48]. Clinical management thus seems to require an intricate and targeted modulation of specific pathways driving secondary cardiac damage. A previous study by Zimmermann et al. reported the underlying molecular mechanisms driving inflammatory processes in the infarct zone [12]. Interestingly, several immune responses were inhibited by the PBMCsec in the non-perfused area. Since we observed improved clinical outcome with the PBMCsec treatment [22,27], inhibition of these pro-inflammatory pathways might represent one way of attenuating secondary tissue damage without causing cardiotoxicity. Surprisingly, genes associated with cardiac muscle contraction were downregulated in the infarcted area when compared with intact myocardium [12], while these genes were induced by the PBMCsec. Zimmermann and colleagues further identified kruppel-like factor 4 (KLF4) and KLF4-dependent signaling as major players in the cardiac ischemic response [12]. In our data set, *KLF4* and KLF4 downstream genes, such as *ACTA1*, *ACTC1*, *DSTN*, and *GATA4*, were not present amongst the genes differentially expressed by the PBMCsec, indicating that the therapeutic effect of the PBMCsec presumably occurs in a KLF4-independent manner. Similarly, insulin signaling was found strongly induced in the border and remote zone of infarcted myocardium compared with healthy controls [12], while our data showed no regulation of insulin signaling by the PBMCsec. These data indicated that the PBMCsec targeted factors and pathways to improve post-AMI cardiac performance in a specific manner rather than modulating the major pathways activated in the course of an ischemic response.

Our analysis led to the identification of several factors involved in heart damage after an AMI, which were significantly upregulated by the PBMCsec application. For example, the *NPPA* gene encodes for atrial natriuretic peptide (ANP), a protein with anti-hypertrophic functions in the heart [49]. Increased *NPPA* expression was detected in human ischemic cardiomyopathy [50] and administering ANP reduced the infarct size and improved the outcome in patients with an AMI [51,52]. Interestingly, *NPPA* was induced in the infarct zone but downregulated in the remote zone by the PBMCsec. Thus, a delicate regulation of *NPPA* might be required to promote post-AMI outcomes. Lynch and colleagues investigated cardiac inflammation caused by *MYBPC3* mutation [53]. Hearts of *MYBPC3*-deficient mice displayed a pronounced pro-inflammatory response with infiltrated M1 macrophages. Furthermore, a systemic state of inflammation was observed. Our results showed that the PBMCsec strongly induced *MYBPC3* with concomitant inhibition of *IFITM3*, *ISG15*, *IFIT1*, and *LYZ* in the infarct zone. Findings reported by Lynch et al. suggest that the PBMCsec might protect from cardiac inflammation by engaging *MYBPC3* and simultaneously downregulating pro-inflammatory genes. In addition to *NPPA* and *MYBPC3*, *MYH6* was among the most upregulated genes by the PBMCsec in the infarcted area. A very recent report by Chen et al. showed that *MYH6* expression was strongly decreased in patients suffering from ischemic cardiomyopathies, such as coronary artery disease, AMI, and heart failure [50]. These data provide a potential explanation for the PBMCsec-mediated increase of *MYH6* expression in the ischemic zone. However, future studies are required to elucidate the functional relevance of decreased *MYH6* levels following ischemic insults and the role of elevated *MYH6* expression by the PBMCsec in cardioprotection against ischemic insults. Our transcriptional analyses further revealed that *ALOX12*/*ALOX15* were downregulated within the border zone but upregulated in the remote zone, suggesting that the PBMCsec induced a distinct gene expression program in perfused, yet hypocontractile, and fully functional myocardium. ALOX12 was implicated in hypoxic preconditioning to reduce cardiac susceptibility to ischemia-reperfusion (IR) injury [54]. In contrast to this finding, pharmacological inhibition of ALOX12 ameliorated myocardial IR injury [55] and ALOX12-dependent signaling triggered an inflammatory response, resulting in exacerbated hepatic IR injury [56]. Conversely, *Alox15* was found downregulated in both AMI and IR [57]. Hence, PBMCsec-mediated downregulation of *ALOX12*/*ALOX15* in the transition zone might protect the myocardium at risk from immunological insults, while *ALOX12*/*ALOX15* upregulation in the remote zone might be involved in the preconditioning of viable myocytes against ischemic events. However, elucidating the exact local effects of PBMCsec-mediated *ALOX* gene regulations merits future investigations. *MMP9* was among the genes most upregulated by the PBMCsec in the transition zone and MMP9 is a well-established instigator of cardiac remodeling post AMI [58]. Whether the anti-fibrotic effects of PBMCsec were caused by modulating *MMP9* expression will be the subject of further studies.

Intriguingly, the PBMCsec exerted anti-inflammatory effects in all tissues investigated except for the transition zone, where a pro-inflammatory chemokine profile was induced. Local upregulation of the chemokine response might favor faster passing of infarct stages and might accelerate healing processes via faster recruitment of leukocytes and macrophages [59,60,61]. Together, these local and tissue-specific events might represent a possible mechanism by which PBMCsec promotes angiogenesis and preserves myocardium at the transition zone.

In addition to the cytoprotective and anti-inflammatory action of the PBMCsec, we also observed an immunological role of the PBMCsec by downregulating *SLA-5* (MHC class I antigen) expression in all tissues investigated. This finding is in line with a previous investigation, where the downregulation of HLA-DR, *HLA-DRA*, *HLA-DBP1*, and *HLA-DQA1* by the PBMCsec in human monocyte-derived dendritic cells correlated with diminished antigen-presenting ability [21]. These data indicated that the PBMCsec tunes down antigen presentation in the myocardium and distal organs, presumably to prevent exacerbated immune responses. Our analysis also led to the identification of several factors with unknown functions in MI to date and, therefore, builds a basis for more in-depth studies in the future.

The earliest works described AMI as a local event exclusively restricted to the non-perfused heart [62]. Ever since then, the concept of tissue affected by an AMI has gradually expanded from the site of ischemia to the entire myocardium and distal organs, and at present, AMI is considered a systemic condition. While several metabolic pathways were reportedly downregulated in the liver following an AMI [12], we detected enrichment of genes implicated in the urea and tricarboxylic cycle (TCA) by the PBMCsec. Interestingly, the TCA cycle metabolites were decreased following hepatectomy with IR [63] and the AMI damage was ameliorated via enhancing TCA enzymatic activities [64]. Inducing TCA genes thus might seem a plausible mechanism by which the PBMCsec exerts distal hepato-protective effects after an AMI. In both liver and spleen, the PBMCsec downregulated several immunoglobulin- and interferon-related genes, such as *IFITs* and *ISG15*. These data indicated that PBMCsec inhibits systemic inflammation following an AMI and that PBMCsec exerts local and distal anti-inflammatory effects. Splenic metabolism was markedly accelerated in patients with an AMI, as evidenced by increased glucose uptake [65], and we observed elevated lipid metabolism by the PBMCsec in the spleen. Intriguingly, the lipid mediator resolvin D1 impaired neutrophil priming in the spleen and the post-AMI heart. Furthermore, resolvin D1 reduced collagen deposition and improved left ventricular function after an MI [66]. We were previously able to demonstrate the presence of anti-inflammatory lipid species in the PBMCsec, such as resolvin D1, D2, D3, and D4 [21]. Therefore, it is tempting to surmise that certain lipid mediators of the PBMCsec are responsible for local and distal anti-inflammatory actions. Future studies are warranted to determine the potential therapeutic effect of secretome-derived resolvin species to improve post-AMI outcomes.

In addition to necrosis, the programmed form of cell death, namely, apoptosis, has long been known to play a major role in AMI [67,68]. Cardiomyocytes and other cells and tissues suffering from insufficient blood supply are prone to undergo ischemia-induced apoptosis. It is conceivable that these cells might also secrete factors with local and distal effects, which is a scenario comparable to the administration of the secretome by stressed PBMCs. However, the therapeutic potential of the inherent secretome might be very limited with regard to AMI, as the cardiac performance of medium-treated animals was compromised, especially after 30 days. Irradiation-induced cell death of PBMCs was identified as an indispensable prerequisite for promoting cardiac regeneration following an AMI [17]. Hence, the intrinsic apoptosis levels seem to be insufficient to prevent post-AMI tissue damage. In addition, the secretory capacity of irradiated PBMCs might exceed that of ischemic cardiomyocytes. These aspects might explain why PBMCsec treatment exerts beneficial effects in addition to those of autologous secretomes generated by apoptotic cells.

In summary, we observed highly tissue-specific effects of the PBMCsec in the context of AMI. Except for the transition zone, the overall effect of PBMCsec was rather anti-inflammatory. Though the immune-suppressive action might be favorable to prevent an exaggerated immune response, diminished immunological actions might predispose for increased risk of infectious disease. As the PBMCsec treatment following AMI is limited to a single dose, this might only represent a side effect confined to the acute phase. Systemically, the PBMCsec affected metabolic processes in liver and spleen. Whether this modulation might include potential adverse events remains to be determined. Whether a single dose or repeated doses of intravenously administering the PBMCsec leads to chronic side effects merits future toxicological and safety studies.

### Limitations

Despite our best efforts, we recognize some limitations of our work. Large animal models, and especially pigs, display a high similarity to the human organism and, therefore, represent a valuable tool to mimic and to study human diseases in a pre-clinical setting. Nonetheless, findings obtained in animal models might not be unreservedly able to be extrapolated to the human system. In addition, our animal model did not consider comorbidities. As comorbidities, such as diabetes, chronic kidney disease, chronic obstructive pulmonary disease, cerebrovascular disease, and peripheral artery disease, are associated with post-AMI in-hospital mortality and other adverse outcomes [69], future studies will be required to test the therapeutic potential of the PBMCsec in AMI models with comorbidities. Though we performed functional assessments and were able to show improved cardiac performance, our study was limited to transcriptional analyses. Delineating the impact of selected genes of interest at the protein level remains the subject of future investigations.

## 5. Conclusions

This pharmacodynamics study identified several putative starting points for the improved treatment of AMI via the application of the secretome of stressed PBMCs. Our transcriptomics data are therefore an important basis for a series of future, more sophisticated in vitro and in vivo pre-clinical experiments, which will be necessary for translation into the clinics.

## 6. Patents

The Medical University of Vienna has claimed financial interest. H.J.A. holds patents related to this work (WO 2010/079086 A1; WO 2010/070105 A1; EP 3502692; European Patent Office application no. 19165340.1).

## Data Availability

Microarray data are available upon request.

## Supplementary Materials

The following supporting information can be downloaded at: https://www.mdpi.com/article/10.3390/biology11010116/s1, Supplementary File S1: Lists of differentially expressed genes, Supplementary File S2: GO terms and associated genes.
